# Novel linear lipopeptide paenipeptin C′ binds to lipopolysaccharides and lipoteichoic acid and exerts bactericidal activity by the disruption of cytoplasmic membrane

**DOI:** 10.1186/s12866-018-1381-7

**Published:** 2019-01-08

**Authors:** Sun Hee Moon, En Huang

**Affiliations:** Department of Environmental and Occupational Health, University of Arkansas for Medical Sciences, 4301 West Markham Street, Little Rock, AR 72205 USA

**Keywords:** Antibiotic, Linear lipopeptide, Paenipeptin C′, Lipopolysaccharide, Lipoteichoic acid, Cytoplasmic membrane

## Abstract

**Background:**

There is an urgent need to develop potent antimicrobials for the treatment of infections caused by antibiotic-resistant bacterial pathogens. Paenipeptin C′ (C8-Pat) is a novel linear lipopeptide recently discovered by our group. The objectives of this study were to determine the time-kill kinetics of paenipeptin C′ against *Pseudomonas aeruginosa* ATCC 27853 and *Staphylococcus aureus* ATCC 29213 and to investigate its mechanism of action.

**Results:**

Paenipeptin C′ was synthesized by solid-phase peptide synthesis and purified by HPLC to homogeneity. Paenipeptin C′ showed concentration-dependent bactericidal activity against *P. aeruginosa* and *S. aureus.* Purified lipopolysaccharides (LPS) from the outer membrane of Gram-negative bacteria and lipoteichoic acid (LTA) from Gram-positive bacteria significantly decreased the antibacterial activity of paenipeptin C′, which indicated that LPS and LTA on cell surfaces are likely the initial binding targets of this antibiotic agent. Moreover, paenipeptin C′ damaged bacterial cytoplasmic membranes, as evidenced by the depolarization of membrane potential and leakage of intracellular potassium ions. Specifically, paenipeptin C′ at 32–64 μg/mL caused a significant membrane potential depolarization in *P. aeruginosa* and *S. aureus*. This antibiotic at 64–128 μg/mL rapidly induced the release of intracellular potassium ions from *P. aeruginosa* and *S. aureus*. Transmission electron microscopy imaging results showed that paenipeptin C′ at bactericidal concentrations perturbed the cell envelopes, leading to the loss of intracellular contents.

**Conclusions:**

Therefore, paenipeptin C' exerts its bactericidal effect through damaging bacterial cytoplasmic membrane.

## Background

Antibiotics have significantly improved public health by curing bacterial infections [1]. However, due to extensive use of antibiotics as therapeutics in human medicine and as growth promoters in food animal production, the period of strong potency for an antibiotic could be relatively short-lived as resistant strains emerged. The global emergence and spread of antibiotic-resistant strains of commonly encountered pathogens has been observed over the past few decades [2, 3]. ESKAPE pathogens, including *Enterococcus faecium*, *Staphylococcus aureus*, *Klebsiella pneumoniae*, *Acinetobacter baumanni*, *Pseudomonas aeruginosa*, and *Enterobacter* species, are recognized antibiotic-resistant bacterial pathogens that can escape the therapeutic effect of most current antimicrobial agents [4, 5]. To overcome this problem, we need to develop new and potent antimicrobial agents against antibiotic-resistant bacteria.

Microorganism-derived natural products and their analogues remain one of the most important sources for novel antibiotics. Microbial peptides, especially lipopeptide antibiotics and their semisynthetic derivatives, are promising candidates for the development of a new generation of antibiotics [6]. Lipopeptide antibiotics generally consist of an N-terminal fatty acyl chain and a linear or cyclic peptide. Natural lipopeptides are synthesized through non-ribosomal peptide synthetases (NRPS) in microbial cells [7]. *Paenibacillus* strains are well known for their ability of producing cyclic lipopeptide antibiotics, such as polymyxins, fusaricidins, and paenibacterin [8–10].

**Fig. 1:**
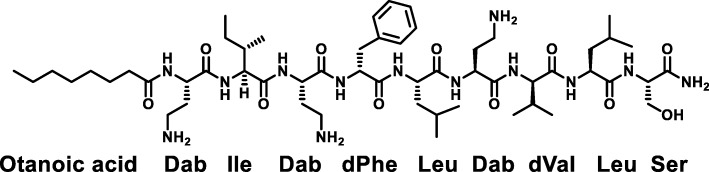
Chemical structure of the synthetic linear lipopeptide paenipeptin C′. Dab stands for 2,4-diaminobutyric acid, which is a positively-charged amino acid

Paenipeptin C' is a novel synthetic linear lipopeptide antibiotic based on the natural products from *Paenibacillus* sp. OSY-N [11]. This antibiotic consists of an N-terminal octanoyl lipid tail and a 9-resisdue linear peptide (Fig. 1). Other examples of reported synthetic linear lipopeptide antibiotics include tridecaptin A1 and linear battacin [12, 13]. Linear lipopeptides and their analogues can be chemically synthesized using standard solid-phase peptide synthesis. Our previous research demonstrated that paenipeptin C′ showed potent antimicrobial activity with minimum inhibitory concentration of 0.5–4.0 μg/mL for Gram-negative strains and 0.5–32 μg/mL for Gram-positive strains [11]. The objectives of this study were to determine the bactericidal activity of paenipeptin C′ by time-kill assays and to investigate its mechanism of antibacterial action.

## Methods

### Paenipeptin C′ (C8-pat) preparation

Paenipeptin C′ was synthesized by solid-phase peptide synthesis (SPPS) and purified by HPLC to homogeneity (> 95% purity) through custom peptide service (Genscript Inc., Piscataway, NJ). The accurate mass of paenipeptin C′ was determined using liquid chromatography mass spectrometer (Agilent 6210 Time-of-Flight, Agilent Technologies, Santa Clara, CA). The theoretical mass of paenipeptin C′ [M + H]^+^ ion is 1116.7510 *m/z*, which is consistent with the measured mass of 1116.7518 *m/z*.

### Bacterial strains, growth media, and culture conditions

*P. aeruginosa* ATCC 27853 and *S. aureus* ATCC 29213 were obtained from American Type Culture Collection (ATCC, Manassas, VA). Both bacterial strains were cultured in tryptic soy broth (TSB; Becton Dickinson, Sparks, MD) or tryptic soy agar (TSA) at 37 °C aerobically.

### Time-kill kinetic assays

Time-kill kinetic assays were used to determine the bactericidal effect of paenipeptin C′ against *P. aeruginosa* ATCC 27853 and *S. aureus* ATCC 29213 as described previously [13]. An overnight bacterial culture of *P. aeruginosa* or *S. aureus* was diluted (1/100 dilution) into tryptic soy broth. One mL of the diluted *P. aeruginosa* cells (approximately 10^6^ CFU/mL) was mixed with an equal volume of paenipeptin C' solution in TSB at a final concentration of 8, 16, or 32 μg/mL. Similarly, paenipeptin C' was added to the diluted *S. aureus* cell suspension (approximately 10^6^ CFU/mL) at a final concentration of 16, 32, or 64 μg/mL. The mixtures were incubated at 37 °C and the number of surviving cells was determined by spread-plating on tryptic soy agar at 0, 2, 4, 6, and 24 h. The colonies on agar plates were counted after 24 h of incubation at 37 °C. The detection limit of quantification in this assay was 10 CFU/mL.

### Effect of lipopolysaccharides (LPS) and lipoteichoic acid (LTA) on antimicrobial activity

Purified LPS from the outer membrane of *E. coli* (Sigma, St. Louis, MO) or LTA from *S. aureus* (Sigma) was used to measure the possible interaction between paenipeptin C' and LPS or LTA according to previous studies [14, 15]. A stock solution of LPS or LTA was prepared in sterilized water, at 1 mg/mL, and kept at − 20 °C. LPS was added to the *P. aeruginosa* ATCC 27853 cell suspension (10^6^ CFU/mL) at a final concentration of 0, 10, 25, or 100 μg/mL. Then paenipeptin C′ was added to a final concentration of 16 μg/mL. The mixtures were incubated at 37 °C for 1 h. The number of surviving cells after treatment was determined by spread-plating on tryptic soy agar. Polymyxin B which binds to LPS was used at 2 μg/mL as a positive control. Similarly, the effect of LTA at various concentrations (0, 10, 25, or 100 μg/mL) on the antibacterial activity of paenipeptin C′ at 32 μg/mL was tested against a Gram-positive bacterium, *S. aureus* ATCC 29213. The inoculum size, incubation conditions, and the methods for surviving cell determination were similar to the procedures as described above in the LPS binding experiment. Nisin, a cationic lantibiotic antimicrobial peptide with known binding ability to LTA [15], was used at 16 μg/mL as a positive control.

### Membrane potential depolarization

Cytoplasmic membrane depolarization after paenipeptin C′ treatment was measured using a fluorescent probe DiSC_3_(5) as described by Huang and Yousef [14]. An overnight bacterial culture of *P. aeruginosa* ATCC 27853 or *S. aureus* ATCC 29213 was diluted 100 times into tryptic soy broth. The diluted cells were incubated at 37 °C with agitation at 200 rpm for approximate 5 h. The resulting cells at early exponential phase were centrifuged at 3660×*g* at 4 °C for 10 min and washed two times with 5 mM HEPES buffer (Sigma) containing 5 mM glucose (buffer A, pH 7.2). The washed *S. aureus* cells were resuspended in buffer A to an OD_600nm_ of 0.05. Conversely, *P. aeruginosa* cells were resuspended to an OD_600nm_ of 0.05 in a solution composed of buffer A and 0.2 mM EDTA (buffer B); the chelating agent EDTA in buffer B can facilitate the uptake of the DiSC_3_(5) probe into cells of Gram-negative bacteria. Then the cell suspension (20 mL) was mixed with 10 μL of 100 μM fluorescent probe DiSC_3_(5). The mixture was incubated for 15 min at room temperature in dark to allow the uptake of the DiSC_3_(5) probe. After incubation, KCl was added to both cell suspensions at a final concentration of 100 mM. Aliquots (90 μL) of the cell suspension were transferred to wells of a black NBS microplate (Corning Inc., Corning, NY), followed by adding paenipeptin C' at various final concentrations (0–128 μg/mL). A change in fluorescence due to membrane depolarization was recorded using a Cell Imaging Multimode Reader (Cytation 3, BioTek, Winooski, VT) at excitation and emission wavelengths of 493 nm and 516 nm, respectively.

### Intracellular potassium release assay

Potassium ions released from paenipeptin C'-treated bacterial cells were determined using a K^+^-sensitive fluorescence probe (PBFI; Invitrogen) [14]. Bacterial cells of *P. aeruginosa* ATCC 27853 or *S. aureus* ATCC 29213 were prepared, washed and resuspended in HEPES buffer with 5 mM glucose (buffer A, pH 7.2) as described earlier, and the cell density was adjusted to an OD_600nm_ of 0.3. The potassium-sensitive probe, PBFI, was added to the cell suspensions at a final concentration of 2 μM. Ninety μL of the cell suspension in buffer A were added to wells of a black NBS microplate, followed by adding paenipeptin C′ at various final concentrations (0–128 μg/mL). The fluorescence reading was monitored by a Cell Imaging Multimode Reader (Cytation 3, BioTek) at an excitation wavelength of 346 nm and an emission wavelength of 505 nm.

### Transmission electron microscopy (TEM)

An overnight bacterial culture of *P. aeruginosa* ATCC 27853 or *S. aureus* ATCC 29213 was 1/10 diluted in sterile saline and treated by paenipeptin C′ at 16 μg/mL or 32 μg/mL, respectively, at 37 °C for 2 h. The treated cells were washed using Hank’s Balanced Salt Solution (HBSS) and pelleted by centrifugation at 3660×*g* at 4 °C for 10 min. The washed cells were fixed with 2.5% glutaraldehyde in 0.1 M sodium cacodylate buffer for 20 min. After washing in cacodylate buffer, cells were post-fixed for 30 min in 1% osmium tetroxide (Electron Microscopy Sciences, Hatfield, PA) with 0.8% potassium hexacyanoferrate (III) (Sigma) in cacodylate buffer. The cells were treated with 1% tannic acid in molecular grade water before dehydrating in a graded ethanol series followed by propylene oxide. The dehydrated cells were embedded in araldite/Embed 812 resins (Electron Microscopy Sciences) and cured for 48 h at 60 °C. Thin sections (50 nm) were collected on 150 mesh copper grids and post-stained with uranyl acetate and lead citrate for imaging at 80 kV with a Technai F20 TEM (Fei, Hillsboro, OR).

### Statistical analysis

For LPS/LTA binding experiments, the bacterial population counts were analyzed and compared. For membrane potential depolarization and potassium release assays, the changes of fluorescence before and after adding paenipeptin C' were recorded and compared. All experiments were conducted at least three times independently. The above data were analyzed using Analysis of Variance (ANOVA) and Tukey’s honest significant difference (HSD) in SPSS Statistics (version 24; SPSS Inc., Chicago, IL).

## Results

### Time-kill kinetics of paenipeptin C′

**Fig. 2:**
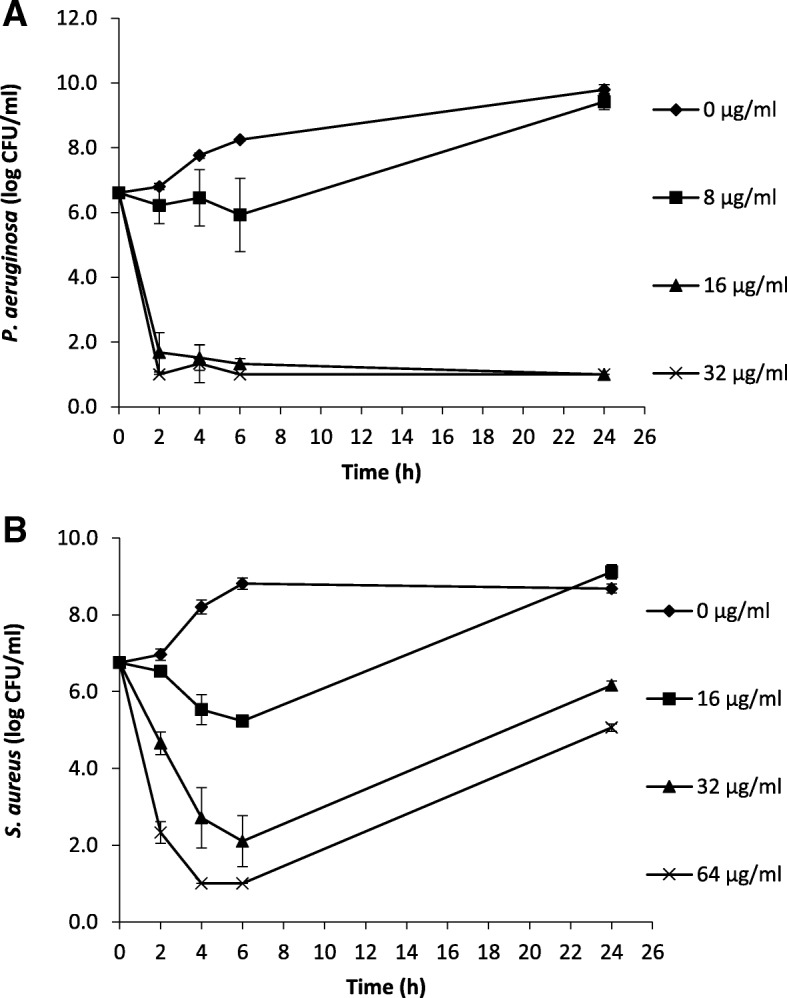
Concentration-dependent bactericidal activity of lipopeptide paenipeptin C′ against (**a**) *Pseudomonas aeruginosa* ATCC 27853 and (**b**) *Staphylococcus aureus* ATCC 29213

Time-kill assays are the appropriate approach to determine the bactericidal effect of an antimicrobial agent. Time-kill kinetics of paenipeptin C′ was determined using two reference strains, *P. aeruginosa* ATCC 27853 and *S. aureus* ATCC 29213. As shown in Fig. 2a, the non-treated *P. aeruginosa* cells grew rapidly and reached 9.8 log after 24 h of incubation at 37 °C. Paenipeptin C′ at low concentration (8 μg/mL) increased the lag time by inhibiting the growth of *P. aeruginosa* in the first 6 h but the final cell population reached a similar level to the non-treated control. Paenipeptin C′ treatment at 16 μg/mL and 32 μg/mL rapidly inactivated 4.9 log and 5.6 log *P. aeruginosa* cells, respectively, within 2 h; the viable cell counts were below the detection limit of 10 CFU/mL at 24 h.

As shown in Fig. 2b, the non-treated *S. aureus* cells grew from 6.8 log to 8.8 log within 6 h and maintained at a similar level at 24 h. Paenipeptin C' also showed a concentration-dependent bactericidal effect against *S. aureus*. This antibiotic at 16 μg/mL resulted in 1.5 log decrease of *S. aureus* at 6 h but a regrowth was observed at 24 h. Paenipeptin C′ at 32 μg/mL and 64 μg/mL produced a steady decline of cell populations from 6.8 log before treatment to 2.1 log and 1.0 log at 6 h, respectively. However, the cell population rebounded to 6.2 log and 5.1 log at 24 h in the presence of paenipeptin C' at 32 μg/mL and 64 μg/mL, respectively. A higher concentration or more frequent dosing of paenipeptin C′ might be required to completely suppress the regrowth of the bacterium. A similar concentration-dependent killing and regrowth kinetics has been reported when *Escherichia coli* was treated by ciprofloxacin [16].

### Effect of lipopolysaccharides (LPS) or lipoteichoic acids (LTA) on antimicrobial activity

**Fig. 3:**
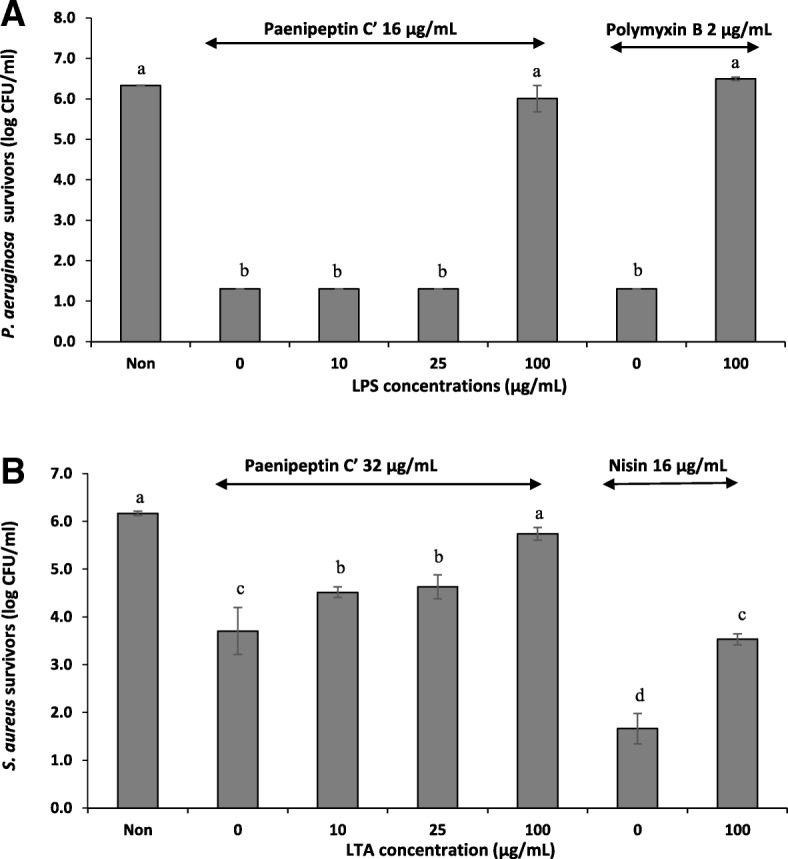
Impact of lipopolysaccharides (LPS) and lipoteichoic acid (LTA) on antimicrobial activity of paenipeptin C′ against (**a**) *Pseudomonas aeruginosa* ATCC 27853 and (**b**) *Staphylococcus aureus* ATCC 29213. The nontreated cells (Non) without antibiotics and LPS/LTA were used as control groups. Means with different letters are significantly different between groups (*p* < 0.05)

The lipopolysaccharides (LPS) from Gram-negative bacteria affected the antimicrobial activity of paenipeptin C′ against *P. aeruginosa* ATCC 27853. Paenipeptin C′ at 16 μg/mL resulted in 5 log reduction of *P. aeruginosa* after 1 h treatment. LPS at low concentrations (10 and 25 μg/mL) had no effect on the antimicrobial activity of paenipeptin C′. However, at 100 μg/mL, LPS completely neutralized the bactericidal activity of paenipeptin C′ (Fig. 3a). Similarly, the positive control, polymyxin B, which is well known to bind to LPS, showed little activity in the presence of 100 μg/mL LPS. Therefore, similar to polymyxin B, the cationic lipopeptide paenipeptin C′ showed affinity to the negatively-charged LPS, suggesting that LPS on the outer membrane of Gram-negative bacteria is likely the initial binding target of paenipeptin C′.

For Gram-positive bacteria, the negatively-charged LTA showed a dose-dependent reaction on the activity of paenipeptin C′ against *S. aureus* ATCC 29213. As showed in Fig. 3b, purified LTA at 100 μg/mL significantly reduced the antibacterial activity of paenipeptin C′ against *S. aureus*. Nisin, a compound known to bind to LTA also showed a significant decrease in its activity in the presence of LTA. These results also suggested that LTA on the cell surface of Gram-positive bacteria is likely the initial target of paenipeptin C′ through electrostatic interaction.

### Depolarization of cytoplasmic membrane by paenipeptin C'

A membrane potential-sensitive fluorescent probe DiSC_3_(5) was used to evaluate the effect of paenipeptin C′ on the integrity of the cytoplasmic membranes of *P. aeruginosa* ATCC 27853 and *S. aureus* ATCC 29213. After incubation with bacterial cells, the DiSC_3_(5) probe accumulated within the healthy hyperpolarized cytoplasmic membrane and become self-quenched. If the integrity of cytoplasmic membrane was disrupted and the membrane potential was dissipated by membrane-active compounds, the DiSC_3_(5) probe was released from the damaged membranes accompanying with an increase of fluorescence.

**Fig. 4:**
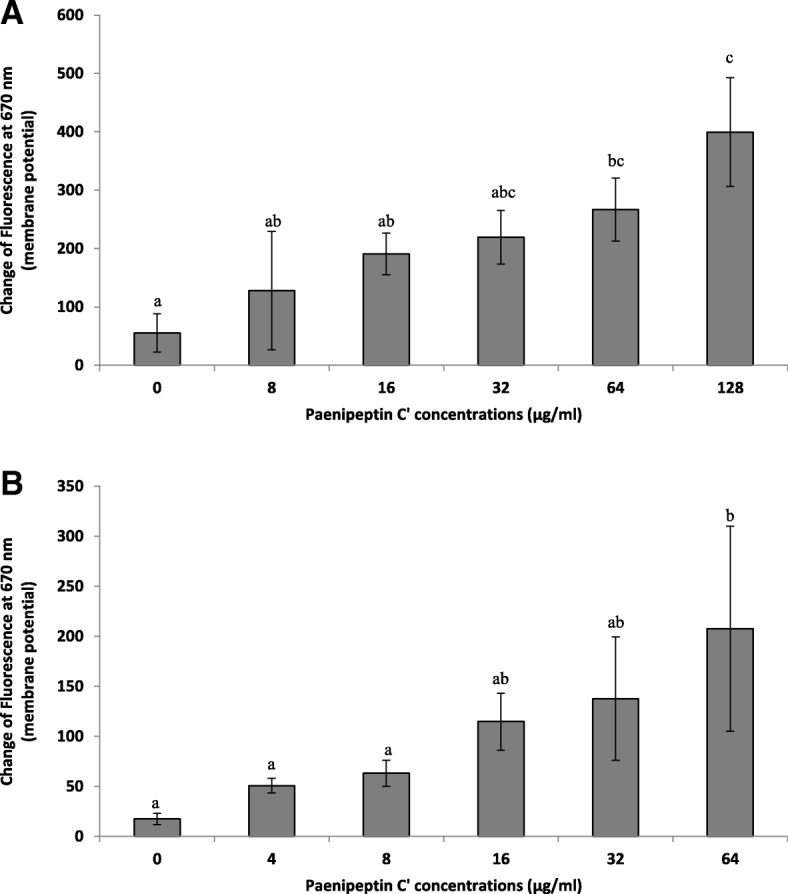
Changes in bacterial membrane potential in the presence of paenipeptin C′ as determined using a membrane potential-sensitive fluorescent probe, DiSC_3_(5). **a** *Pseudomonas aeruginosa* ATCC 27853 (**b**) *Staphylococcus aureus* ATCC 29213. Means with different letters are significantly different between groups (*p* < 0.05)

As shown in Fig. 4a, the treatment of paenipeptin C′ at 64 μg/mL significantly enhanced the fluorescence signal, which suggested that paenipeptin C′ permeabilized the cell membrane of *P. aeruginosa*. Similarly, paenipeptin C′ at 64 μg/mL caused a significant membrane potential disturbance on *S. aureus* (Fig. 4b). These data suggested that paenipeptin C′ at bactericidal concentrations compromised the integrity of the cell membrane and dissipated the membrane potential of *P. aeruginosa* and *S. aureus*.

### Paenipeptin C' triggered the release of intracellular potassium ions

**Fig. 5:**
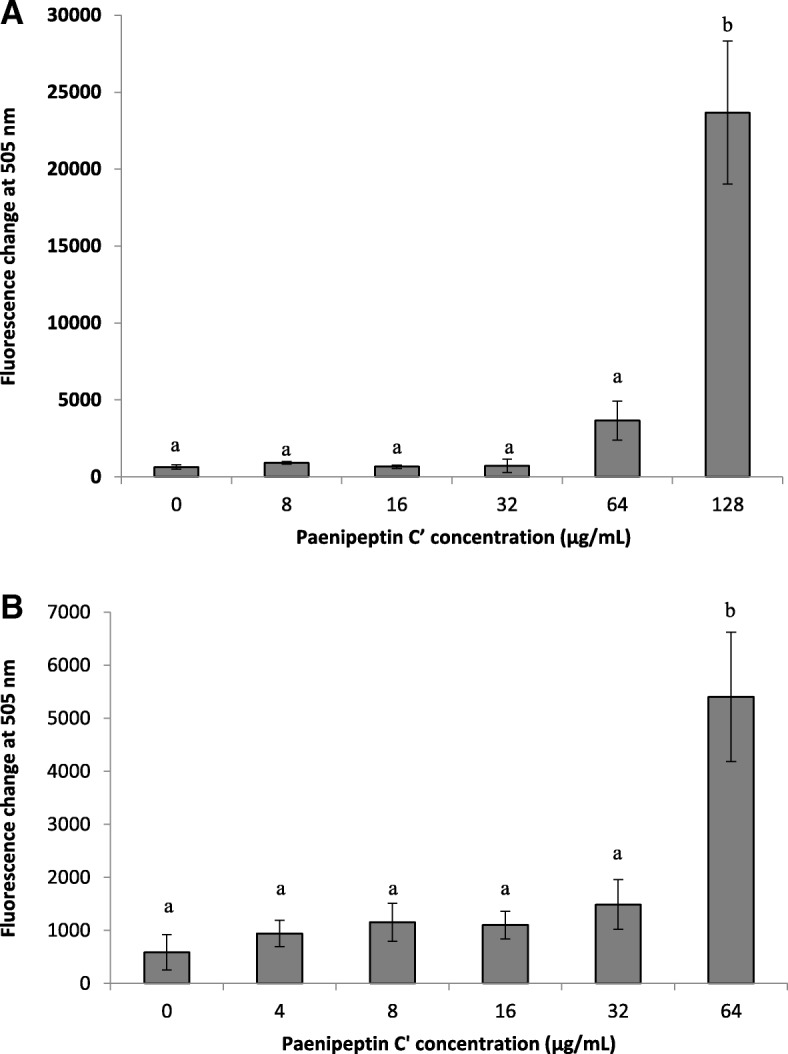
Release of intracellular K^+^ in the presence of paenipeptin C′ as detected using a potassium-sensitive fluorescent probe, PBFI. **a** *Pseudomonas aeruginosa* ATCC 27853 (**b**) *Staphylococcus aureus* ATCC 29213. Means with different letters are significantly different between groups (*p* < 0.05)

To further investigate the membrane damage caused by paenipeptin C′, the release of intracellular potassium ions from paenipeptin C′ treated cells was measured using a potassium-sensitive probe, PBFI. At low concentrations (< 32 μg/mL), paenipeptin C′ did not induce significant potassium ion leakage from *P. aeruginosa* ATCC 27853 or *S. aureus cell* ATCC 29213*.* As shown in Fig. 5a, paenipeptin C′ at 128 μg/mL significantly increased extracellular K^+^ concentration in the treated *P. aeruginosa* cells. Similarly, significant potassium ion leakage from *S. aureus* ATCC 29213 cells was observed after treatment with paenipeptin C' at 64 μg/mL (Fig. 5b).

### Transmission electron microscope (TEM) observation

**Fig. 6:**
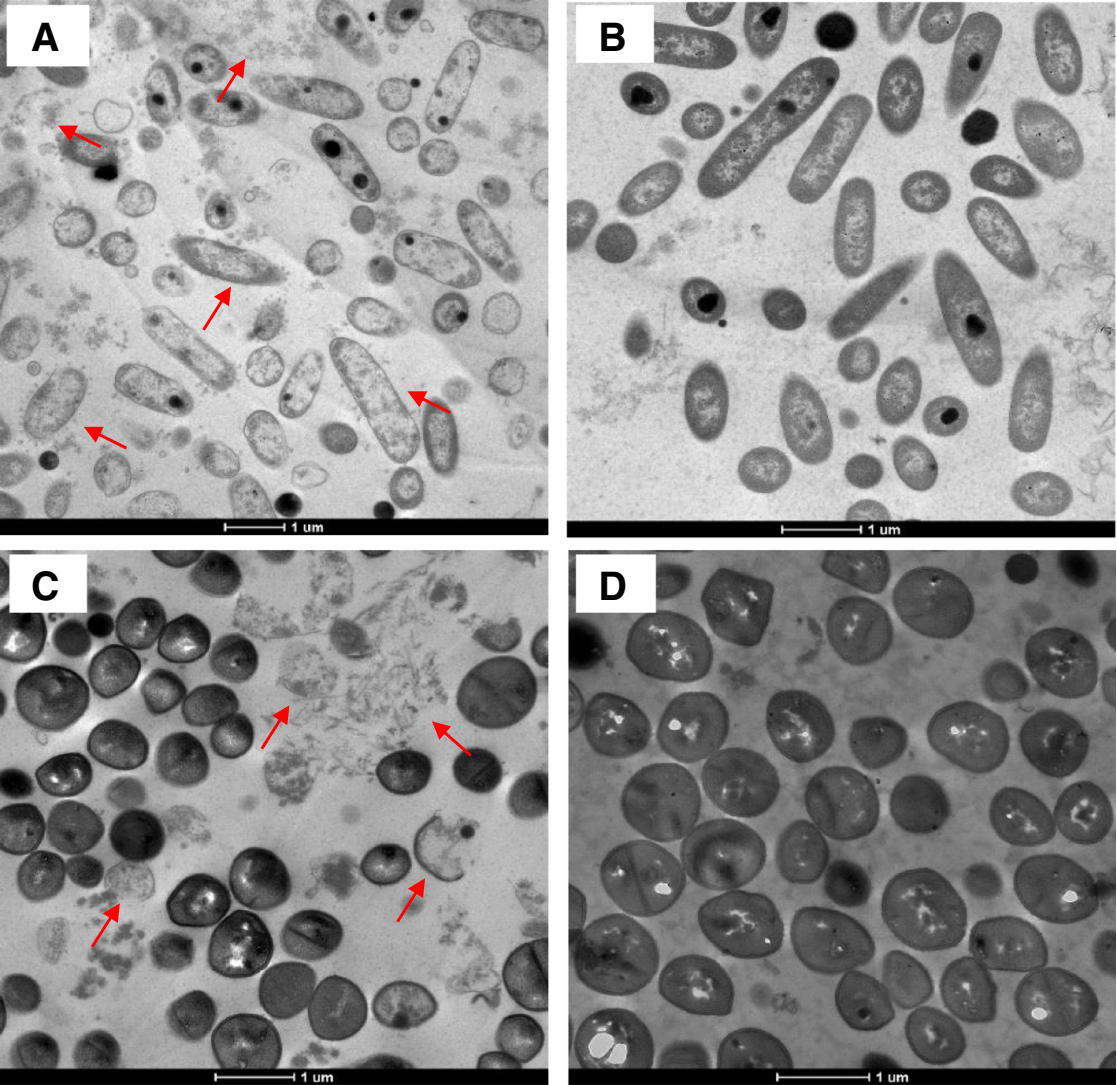
Examination of the cell morphology change after paenipeptin C′ treatment using transmission electron microscopy (TEM). **a** *Pseudomonas aeruginosa* ATCC 27853 cells were treated by paenipeptin C′ at 16 μg/mL for 2 h. **b** Non-treated *P. aeruginosa* ATCC 27853 cells. **c** *Staphylococcus aureus* ATCC 29213 cells were treated by paenipeptin C' at 32 μg/mL for 2 h. **d** Non-treated *S. aureus* ATCC 29213 cells

The morphology and intracellular alterations in bacterial cells after paenipeptin C′ treatment were directly observed by TEM to better understand the antibacterial mechanism of paenipeptin C′. As shown in Fig. 6a, substantial morphology changes occurred when the *P. aeruginosa* ATCC 27853 cells were treated with paenipeptin C′ at 16 μg/mL for 2 h. Conversely, the non-treated *P. aeruginosa* cells displayed the typical Gram-negative rod-shaped structures with intact membrane and high density cytoplasm (Fig. 6b). Similarly, *S. aureus* ATCC 29213 cells treated with paenipeptin C' at 32 μg/mL for 2 h exhibited abnormalities, including disappearance of cell walls, disruption of cell membranes, and even leakage of intracellular contents (Fig. 6c). In contrast, the non-treated *S. aureus* cells retained the normal cell morphology, showing intact cell walls, well-defined membranes, and homogeneous electron density in the cytoplasm (Fig. 6d).

## Discussion

Many cationic peptide antibiotics are able to depolarize the cytoplasmic membrane of their target bacteria, resulting in cell death [14, 15]. We have previously determined that an analogue of paenipeptin C′ (analogue 17), which substitutes the C_8_ fatty acyl chain in paenipeptin C′ with a 3-cyclohexylalanyl group and consists of more hydrophobic amino acid residues, compromised the integrity of bacterial membranes [17]. Paenipeptin C′ showed potent activity against tested Gram-negative and Gram-positive bacteria, including *P. aeruginosa* ATCC 27853 and *S. aureus* ATCC 29213. This study explored the mechanism of action of this broad-spectrum antimicrobial lipopeptide. Paenipeptin C′ consists of three positively-charged amino acids in the peptide chain. The cationic property and the hydrophobicity of the lipid chain in paenipeptin C′ are believed to contribute to the potent antibacterial activity of this compound. The results obtained in this study showed a good correlation between the antimicrobial properties and the membrane-active activities of paenipeptin C′ against *P. aeruginosa* and *S. aureus*. Paenipeptin C′ showed a strong binding activity to both LPS and LTA, the negatively-charged components on the cell surface of Gram-negative and Gram-positive bacteria, respectively. Torcato et al. reported that the cationic peptides, BP100 analogues, were able to bind and neutralize both LPS and LTA [18]. Moreover, paenipeptin C′ depolarized the cytoplasmic membrane and triggered the release of intracellular contents such as potassium ions. Similarly, paenibacterin, a natural cyclic cationic lipopeptide, also targeted cytoplasmic membranes [14]. The TEM images further confirmed that paenipeptin C′ caused a great degree of membrane damage to the treated Gram-negative and Gram-positive cells.

## Conclusion

Taken together, our findings indicated that the antibacterial effect of paenipeptin C′ was likely due to direct alterations of the structure of cell membrane which may give rise to loss of cell viability. However, these findings do not rule out the existence of additional intracellular targets or additional bactericidal mechanisms exerted by paenipeptin C′. Macromolecular biosynthesis assays could be used in future studies to determine the influence of paenipeptin C' on incorporation of precursors into DNA, RNA and proteins.

## Abbreviations

HBSS: Hank’s Balanced Salt Solution
LPS: Lipopolysaccharides
LTA: Lipoteichoic acid
NRPS: Non-ribosomal peptide synthetases
SPPS: Solid-phase peptide synthesis
TEM: Transmission electron microscopy
TSB: Tryptic soy broth
